# Fat Mass Influences Femur Bone Strength and Geometry Parameters, but Not Bone Mineral Density, in Autoimmune Diabetes: A Pilot Study

**DOI:** 10.1002/dmrr.70149

**Published:** 2026-03-19

**Authors:** Renata Risi, Rocco Amendolara, Vahideh Abedi, Francesco De Vita, Federica Barbaro, Angela Balena, Mikiko Watanabe, Daniela Luverà, Angelo Lauria Pantano, Luca D'Onofrio, Raffaella Buzzetti, Ernesto Maddaloni, AD_OWOB study group

**Affiliations:** ^1^ Department of Experimental Medicine Sapienza University of Rome Rome Italy; ^2^ Diabetology Unit San Camillo Forlanini Hospital Rome Italy; ^3^ UOC Diabetologia AOU Policlinico Umberto I Rome Italy

**Keywords:** autoimmune diabetes, bone mineral density, bone strength, fat mass, hip structural analysis

## Abstract

**Background:**

Autoimmune diabetes (AD) increases fracture risk. Overweight worsens bone health despite increasing bone mineral density (BMD). This study examines how adiposity influences BMD and Dual X‐Ray Absorptiometry (DXA)‐derived parameters of bone geometry and strength in AD.

**Methods:**

DXA scan was used to assess body mass composition, BMD, trabecular bone score (TBS), and hip structural analysis (HSA)‐derived parameters at narrow neck (NN), intertrochanteric (IT) and femur shaft (FS) in 52 normal weight adults with AD and 51 with AD and overweight. Multilinear regression models were used to adjust the associations for age, sex, BMI, HbA1c%, lean mass % and physical activity.

**Results:**

Total Fat% was negatively associated with markers of bone strength and geometry at different femur sites, including cross‐sectional moment of inertia (CSMI) and section modulus (Z) at IT site, cross‐sectional area (CSA), CSMI and Z at the FS site and CSMI, buckling ratio (BR) and Z at the NN site, after adjustment for confounders. Visceral adipose tissue emerged as a negative independent predictor of BR at IT and NN. Lean mass % was positively associated with TBS, CSA, CSMI and Z at the IT and FS sites. Negative predictors of bone health were female sex and age.

**Conclusion:**

In adults with AD, excess fat mass, including visceral adiposity, is independently associated with impaired hip bone strength and geometry at the femur shaft. These findings highlight the limitations of BMD alone in evaluating bone health in people with AD and overweight, emphasising the negative skeletal effects of adiposity.

## 1 Introduction

People affected by autoimmune diabetes (AD) show a 6–9 times increased risk of bone fractures compared with people without diabetes [1, 2, 3]. Indeed, people with AD suffer from both reduced bone mineral density (BMD) [4, 5, 6, 7], and poor bone quality [8, 9, 10, 11, 12]. Several factors have been shown to worsen bone health in people with AD, including the presence of classical chronic complications [13, 14, 15, 16, 17], sustained hyperglycaemia, oxidative stress and inflammation [18, 19, 20, 21, 22, 23, 24, 25, 26, 27, 28]. Furthermore, insulin deficiency can impair osteoblast function, leading to decreased bone formation [29, 30], with low C peptide being associated with poorer bone quality in AD [31].

Fat mass excess is considered an emerging risk factor for poor bone health. Adipose tissue acts as an endocrine organ that releases adipokines such as leptin, adiponectin and TNF alpha, promoting bone resorption and inhibiting bone formation [32]. Free fatty acid (FFA) overflow from visceral adipose tissue causes lipotoxicity in osteoblasts, impairing their proliferation and function [33]. Moreover, emerging evidence has shown a detrimental role of increased marrow adipose tissue on bone health and strength [34].

Although AD has been traditionally considered a disease affecting lean people, a dramatical increase in the prevalence of overweight and obesity among people with AD has been documented [35, 36], resulting in novel immunometabolic issues likely further increasing the burden of the disease and impacting on AD outcomes. Recent data have shown that fat excess in people with AD is associated with diabetes‐related complications and disease severity, even differently from other contexts such as type 2 diabetes [37, 38, 39]. Therefore, there is the need of novel data clarifying the relationship between fat mass and bone health in AD.

In this study, we primarily aimed at evaluating whether BMD, bone geometry and strength differ between overweight and normal‐weight people with AD and, secondarily, at investigating the relationships of fat mass and distribution with BMD, bone geometry and strength in AD.

## 2 Methods

### 2.1 Study Design and Population

In this cross‐sectional study, we consecutively enroled people with AD referring to the Diabetes Unit of Policlinico Umberto I General Hospital, Rome, Italy from February 2024 to February 2025. Inclusion criteria were: (1) male with age > 18 and < 80 years old; women in premenopausal status (2) willingness to participate in the study; (3) diagnosis of AD verified by a previous diagnosis of diabetes mellitus and a record of at least one positive pancreatic autoantibody among: glutamic acid decarboxylase, islet tyrosine phosphatase 2, zinc transporter 8 [40]. Exclusion criteria were: clinically relevant bone disease other than osteoporosis, severe psychiatric illnesses, end‐stage renal disease, renal dialysis, hepatic cirrhosis, active cancer of any type, and chronic treatment with corticosteroids and other medications that may affect bone health, including antiresorptive (bisphosphonates, denosumab) or bone‐anabolic agents (teriparatide, romosozumab).

### 2.2 Data Collection

At the time of enrolment, demographic data, medical history, and biochemical information as well as blood samples were collected from each participant. Serum samples were subsequently aliquoted and stored at − 80°C prior to assay. Data regarding diabetes history, smoking habit, physical activity, co‐morbidities, biochemistry (glycated haemoglobin, total cholesterol, triglycerides, high‐density lipoprotein cholesterol, low‐density lipoprotein cholesterol, serum creatinine, estimated glomerular filtration rate (eGFR), and urine albumin) were collected from clinical records. Physical activity was assessed from a questionnaire on the number of hours spent on sports per week (organised sports plus leisure‐time activity). Patients were classified as physically active if the number of hours spent on sports were ≥ 3.5 per week. Anthropometric measures were taken in fasting condition wearing light clothing and no shoes. Body mass index (BMI) was calculated as body weight in kilogrammes divided by height in squared metres (kg/m^2^). Normal weight (NW) was defined for BMI < 25 kg/m^2^ and overweight (OW) for BMI ≥ 25 kg/m^2^.

### 2.3 Dual X‐Ray Absorptiometry (DXA)

All patients underwent Dual X‐Ray Absorptiometry (DXA) scan QDR Discovery Acclaim, (Hologic Inc., Waltham, MA) in fasting condition wearing light clothing and no shoes. We collected the following parameters: Total Fat Mass Percentage (Tot Fat%), Total Soft Lean Mass Percentage (Total Lean%), Visceral Adipose Tissue Mass (VAT mass, kg), BMD at the lumbar spine, total hip, and left femoral neck. All scans were administered by a trained technician using standardized procedures recommended by GE Healthcare. BMD was recorded in terms of the absolute mineral content (in g/cm^2^) at various sites.

The integrated software TBS iNsight, version 2.1.2.0, was applied by the same technician to the site‐matched spine scans for the evaluation of TBS, an indirect indicator of microarchitecture and bone quality estimation.

### 2.4 Hip Structure Analysis (HSA)

The HSA software derives the geometry of the load supporting surface by employing a projection principle first described by Martin and Burr [41]. This is a computational algorithm applied to 2‐dimensional projected images of the hip generated from DXA scans following conventional bone mineral analysis [42]. The programme uses the distribution of mineral mass in a line of pixels across the bone axis. Parameters of bone geometry/strength were derived in 3 femur regions (narrow neck, NN; intertrochanteric, IT; femur shaft, FS):

1. Cross‐sectional area (CSA, mm2), defined as the surface area of bone tissue in the cross‐section after excluding soft tissue space, is proportional to conventional bone mineral content in the corresponding cross‐section. In mechanical terms, CSA is an indicator of resistance to loads directed along the bone axis;
2. Cross‐sectional moment of inertia (CSMI, mm4), derived from the integral of the bone mass weighted by the square of distance from the centre of mass. The CSMI is relevant to bending in the plane of the DXA image
3. Section modulus (Z) is an indicator of the strength of the bone to resist bending and torsion
4. Buckling ratio (BR), a mechanical index of wall stability, was calculated as the distance from the centre of mass to the medial or lateral cortex (whichever distance was larger) divided by the estimated average cortical thickness.

### 2.5 Statistical Analysis and Sample Size Considerations

Continuous variables are presented as median [25th–75th percentile] and categorical variables as number and percentages. Shapiro–Wilk test was used to evaluate the parametric distribution of continuous variables. Non‐parametric variables were ln‐transformed as appropriate. Differences in BMD and hip structure parameters between NW and OW people with AD were evaluated separately for men and women. Multivariate regressions were used to test the association between DXA‐derived markers of bone health and adiposity parameters (Total Fat% and VAT mass) in all populations. Factors associated with markers of bone health were retained at the conservative *p*‐value < 0.1 were retained in the model, with Total Lean%, age, sex (male or female), HbA1c%, BMI, and physical activity habit (physically active or not active) forced in the model as pre‐specified confounders. To control for multiple comparisons, we applied the False Discovery Rate (FDR) correction using the Benjamini‐Hochberg procedure [43]. Adjusted *p*‐values (*q*‐values) were computed, and statistical significance was defined as FDR < 0.05. STATA version 17.0 (StataCorp, College Station, TX, USA) was used for data analysis and Prism 8.4 Software for graphical representations.

### 2.6 Ethics

The study was performed in accordance with the Declaration of Helsinki, and the study procedures were approved by the Umberto I ‘Policlinico’ General hospital ethics committee Prot. 0166/2024. All participants signed written informed consent.

## 3 Results

### 3.1 Population Features

The study population was composed of 103 patients, 57 women (55.3%) and 46 men (44.7%). Median age was 46 years [36.3–53.0], BMI 24.9 kg/m^2^ [22.0–28.0], diabetes duration 12.0 years [4.6–28.2]; 30 patients (29.1%) were current smoker, 25 (24.3%) were physically active. Population features divided by gender and BMI class (NW or OW) are summarised in Table 1. Compared with NW, women with OW (*n* = 25, 43.9%) were younger and had higher levels of triglycerides. Compared to NW, men with OW (*n* = 26, 46.4%) had higher levels of HbA1c% and lower level of Vitamin D. No other significant differences in clinical and biochemical features were found between people with NW or OW.

**TABLE 1:** Clinical anthropometric biochemical parameters of study population stratified by gender and BMI class.

	Women			Men		
	Normal weight (*n* = 32)	Overweight (*n* = 25)	*p* value	Normalweight (*n* = 20)	Overweight (*n* = 26)	*p* value
Age, years	52 (38.25–56.75)	44 (37.5–56)	**0.041**	44.5 (33.25–50.75)	41 (33–51)	0.42
Smokers, *n* (%)	10 (31.3%)	5 (20%)	0.56	7 (35%)	8 (30.8%)	0.73
Physically active, *n* (%)	7 (21.9%)	5 (20%)	0.78	6 (30%)	6 (23.1%)	0.75
BMI, kg/m^2^	22.77 (20.97–24.09)	28.98 (26.05–32.4)	**<** **0.001**	22.06 (21.05–23.72)	27.93 (25.96–30.93)	**<** **0.001**
Diabetes duration, years	12 (4.146–33.25)	14 (5.5–23.58)	0.75	12.5 (2.646–30)	8 (3.5–19)	0.22
Glycaemia, mg/dL	139 (114.5–159)	133 (105–196)	0.76	139 (120.8–202.5)	150 (125.3–217.8)	0.96
Hba1c,%	7.76 (6.97–8.56)	7.85 (6.9–9.15)	0.98	7.15 (6.405–7.45)	7.4 (7.09–8.1)	**0.047**
TC, mg/dL	175 (154–189)	170 (137–199)	0.25	153 (147–167)	176 (149–209)	0.06
HDL‐C, mg/dL	75 (60.5–85)	62 (53–77)	**0.031**	51 (46–65.5)	55 (44–63)	0.54
LDL‐C, mg/dL	85 (71.55–103.5)	83.4 (59–110.8)	0.42	85.6 (70.48–97.3)	108.6 (73–129.2)	0.07
TG, mg/dL	59 (48.25–72.75)	81 (60–95)	**0.001**	72.5 (54.5–103.8)	74 (49–107)	0.81
e GFR, mL/min/1.73 m²	98 (89–107)	105 (90.13–113.3)	0.64	99.45 (88.5–117)	98.6 (87.5–114.8)	0.53
Phosphate, mg/dL	4 (3.4–4.6)	3.7 (3.25–4.3)	0.72	2.8 (2.5–3.7)	3.4 (2.8–3.85)	0.73
Ca, mg/dL	9.7 (9.4–10.1)	9.8 (9.25–10.1)	0.43	9.8 (9.4–9.9)	9.7 (9.425–9.95)	0.53
Vitamin D, ng/mL	32 (20–37.5)	32 (21–36)	0.89	49 (25.5–64)	20.5 (17.75–34)	**0.03**
SBP, mmHg	12 0(110–125)	120 (115–120)	0.92	127.5 (110–137.5)	120 (115–130)	0.25
DBP, mmHg	75 (70–80)	70 (70–80)	0.81	80 (71.25–80)	80 (70–80)	0.59
HR, bpm	84 (67–85)	70 (65–78.5)	0.12	84 (68.5–101)	70 (67–86)	0.15
TIDD/proKg, UI/kg	0.32 (0.22–0.5)	0.48 (0.27–0.54)	**0.03**	0.40 (0.27–0.5)	0.40 (0.29–0.52)	0.26
CSII, *n* (%)	9 (28.1%)	10 (40%)	0.26	6 (30%)	12 (46.2%)	0.36
Metformin, *n* (%)	9(28.1%)	10 (40%)	0.43	7 (35%)	12 (46.2%)	0.56
GLP1Ras, *n* (%)	1 (3.1%)	1 (4%)	0.45	1 (5%)	2 (7.7%)	1
SGLT2i, *n* (%)	0	1 (4%)	0.54	1 (5%)	1 (3.8%)	1
DPP4i, *n* (%)	0	0	0.91	1 (5%)	0	0.43
Vitamin D/Calcium supplements, *n* (%)	5 (15.6%)	6 (24%)	0.56	5 (25%)	3 (11.5%)	0.16
Lipid lowering medications, *n* (%)	16 (50%)	12 (48%)	0.87	6 (30%)	15 (57.7%)	0.13
Antihypertensive medications, *n* (%)	11 (34.4)	6 (24%)	0.65	7 (35%)	2 (7.7%)	0.42
Microalbuminuria, *n* (%)	3 (9.4%)	3 (12%)	0.78	3 (15%)	0	**0.06**
Retinopathy, *n* (%)	4 (12.5%)	4 (16%)	0.77	4 (20%)	2(7.7%)	0.1
Neuropathy, *n* (%)	0	0	na	0	0	na
Previous CV events, *n* (%)	0	0	na	0	0	na

*Note:* Data are expressed as *n* (percentage) or median (25th–75th percentile). Differences between groups are significant per *p* < 0.05 level.

Abbreviations: BMI, Body Massi Index; CSII, Continuous subcutaneous insulin infusion; DBP, diastolic blood pressure; DPP4i, Dipeptidyl peptidase 4 inhibitors; eGFR, estimated glomerular filtrate rate; GLP1Ras, Glucagon‐like peptide 1 receptor agonists; HDL‐C, high density lipoprotein cholesterol; HR, heart rate; LDL‐C, low density lipoprotein cholesterol; previous CV events, previous cardiovascular events; SBP, systolic blood pressure; SGLT2i, Sodium‐glucose co‐transporter 2 inhibitors; TC, Total Cholesterol; TG, Triglycerides; TIDD/kg, Total insulin daily dose pro kg.

### 3.2 Differences in Bone Quality, Geometry and Strength Across Gender and BMI Class

In Table 2, DXA and HSA‐related parameters of bone health are summarised and stratified by gender and BMI class. Women with OW showed greater Total Hip BMD compared with those with NW, and higher CSA at the NN and FS sites. On the contrary, BR was lower at the FS site in women with OW compared with NW (Table 2). A similar trend was observed across men. Men with OW showed greater total hip and femur neck BMD compared to those with NW; moreover, they showed significantly higher CSA and CSMI at the NN, IT and FS sites, while NN BR was lower in men with OW compared to those with NW (Table 2).

**TABLE 2:** DXA and HSA‐derived parameters of bone status of the study population stratified by gender and BMI class.

	Women			Men		
	Normalweight (*n* = 32)	Overweight (*n* = 25)	*p* value	Normalweight (*n* = 20)	Overweight (*n* = 26)	*p* value
TBS	1326 (1.283–1.362)	1332 (1.207–1.404)	0.49	1291 (1.251–1.403)	1332 (1.288–1.391)	0.35
Total fat Mass, g	4281 (3742–5310)	7315 (85671–7849)	**< 0.001**	3113 (2.611–3.644)	4736 (3.823–7.325)	**< 0.001**
Total lean Mass, g	9874 (9371–10,752)	12,597 (10.525–14.420)	**< 0.001**	12,144 (10929–13259)	15,436 (14120–17606)	**< 0.001**
Lumbar spine BMD, g/cm²	0.951 (0.863–1042)	1056 (0.894–1.155)	0.27	0.963 (0.8978–1014)	1068 (0.9078–1174)	**0.082**
Femur neck BMD, g/cm²	0.728 (0.679–0.757)	0.780 (0.696–0.874)	**0.071**	0,7442 (0.6965–0.7653)	0.8474 (0.7551–0.9859)	**0.005**
Total hip BMD, g/cm²	0.807 (0.763–0.876)	0.929 (0.815–1.007)	**0.0041**	0.883 (0.8274–0.9371)	1025 (0.8926–1.143)	**0.005**
NN_CSA	2639 (2344–2818)	3105 (2.477–3.309)	**0.026**	3062 (2.891–3.398)	3.59 (3155–4096)	**0.002**
NN_CSMI	2292 (1666–2856)	2578 (2.086–3.228)	0.13	3525 (2.93–4.289)	3968 (3.732–4.696)	**0.012**
NN_BR	11.56 (9714–13.67)	10.84 (9.016–11.9)	**0.061**	12.65 (11.71–13.93)	10.19 (9.139–12.65)	**0.03**
NN_Z	1255 (0.9805–1449)	1436 (1.125–1.617)	0.12	1772 (1.418–1.978)	2003 (1.66–2.273)	0.23
IT_CSA	4717 (3974–4957)	4831 (4.054–5.778)	0.12	5638 (5.243–6.303)	6587 (5.934–8.006)	**0.001**
IT_CSMI	13.25 (10.41–15.05)	12.83 (10.58–17.15)	0.34	19.53 (16.55–22.57)	23.25 (20.57–29.95)	**0.002**
IT_BR	8938 (8.04–10.46)	8376 (7.204–10.67)	0.15	8577 (7.893–9.659)	7.22 (6.713–8.943)	0.15
IT_Z	3924 (3.262–4.577)	4305 (3.115–4.825)	0.76	5247 (4.628–5.74)	6091 (4.945–2.273)	**0.043**
FS_CSA	3509 (3.335–3.91)	4234 (3.68–4.744)	**0.001**	4623 (4.382–4.78)	5472 (4.935–6.122)	**0.001**
FS_CSMI	3.13 (2.607–3.72)	3131 (2.718–4.414)	0.15	4353 (3.853–5321)	5854 (4.904–6.572)	**0.002**
FS_BR	3472 (2.574–3957)	2543 (2.359–2.961)	**0.003**	2761 (2.421–3.259)	2581 (2.335–3.251)	0.52
FS_Z	2053 (1.826–2.388)	2149 (1.886–2.762)	0.13	2919 (2.396–3.045)	3155 (2.491–3.71)	**0.033**
*z* score lumbar spine	−0.4 (−0.95–0.9)	0.55 (−0.3–1.3)	0.71	−0.8 (−1.3 to −0.5)	0 (−1.075–0.9)	0.92
*z* score femur neck	−0.4 (−0.85–0.025)	−0.2 (−1.1–0.65)	0.27	−0.8 (−1.5–0.2)	0.2 (−1.1–0.875)	0.62
*z* score total hip	−0.6 (−1025–0.05)	0.3 (−0.7–1)	0.35	−0.8 (−1.075–0.3)	0.25 (−0.9–1)	0.12

*Note:* Data are expressed as *n* (percentage) or median (25th–75th percentile). Differences between groups are evaluated via independent *t*‐test for unpaired values.

Abbreviations: FS_BR, Buckling Ratio at the femur shaft; FS_CSA, cross‐sectional area at the femur shaft; FS_CSMI, cross‐sectional moment of inertia the femur shaft; FS_Z, section module at the femur shaft; Differences between groups are evaluated via independent *t*T‐test for unpaired values; IT_BR, Buckling Ratio at the intertrochanteric region; IT_CSA, cross‐sectional area at the intertrochanteric region; IT_CSMI, cross‐sectional moment of inertia at the at the intertrochanteric region; IT_Z, section module at the intertrochanteric region; NN_BR, Buckling Ratio at the narrow neck; NN_CSA, cross‐sectional area at the narrow neck; NN_CSMI, cross‐sectional moment of inertia at the narrow neck; NN_Z, section module at the narrow neck; TBS, Trabecular bone score.

### 3.3 Predictors for BMD

In Table S1 and S3, the standardized beta coefficients and *p*‐values of the multivariate regressions with the BMD‐related outcomes and TBS as dependent variables are reported. In the whole population, Total Fat% and VAT mass were not significantly associated with BMD at any site, as well as with TBS (Tables S1 and S3). To note, in the fully adjusted model, Total Lean%, HbA1c% and physical activity did not show any association to BMD; conversely, BMI was positively associated with total hip (Adj *β* coefficient = 0.605 *p* < 0.001 in Table S1 and Adj *β* coefficient = 0.486 *p* < 0.001 in Table S3), femur neck hip (Adj *β* coefficient = 0.456 *p* < 0.001 in Table S1 and Adj *β* coefficient = 0.362 *p* < 0.001 in Table S3) and lumbar spine BMD (Adj *β* coefficient = 0.652 *p* < 0.001 in Table S1 and Adj *β* coefficient = 0.437*p* < 0.001 in Table S3); age was inversely related to total hip (Adj *β* coefficient = −0.272 *p* = 0.041 in Table S3) and femur neck hip (Adj *β* coefficient = −0.202 *p* = 0.023 in Table S1 and Adj *β* coefficient = −0.314 *p* = 0.030 in Table S3). Finally, male gender was directly associated with total hip BMD (Adj *β* coefficient = 0.225 *p* = 0.023 in Table S1). Independent predictors for a better TBS were higher lean mass (Adj *β* coefficient = 0.280 *p* = 0.023 in Table S3), male sex (Adj *β* coefficient = −0.359 *p* = 0.021 in Table S1) and younger age (Adj *β* coefficient = −0.456 *p* = 0.041 in Table S1).

### 3.4 Predictors for Hip Strength Analysis‐Derived Parameters

**FIGURE 1:**
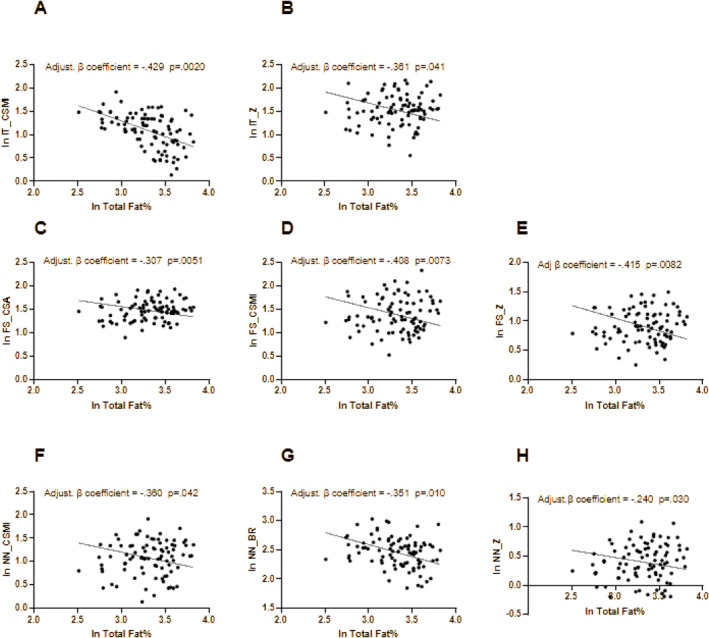
(A–H). Multivariate regressions between Total Fat% and HSA‐derived parameters. Dependent variables: IT_CSMI (A), IT_Z (B), FS_CSA (C), FS_CSMI (D), FS‐Z (E), NN_CSMI (F), NN_BR (G), NN_Z (H). Variables tested in the model: total lean%, age, gender, BMI, HbA1c%, physical activity. Factors associated with markers of bone health at the conservative *p*‐value < 0.1 were retained in the model. BR, buckling ratio; CSA, cross‐sectional area; CSMI, cross‐sectional moment of inertia; FS, femur shaft site; IT, Intertrochanteric site; NN, narrow neck site; Total fat%, total fat percentage; Z, section module.

**FIGURE 2:**
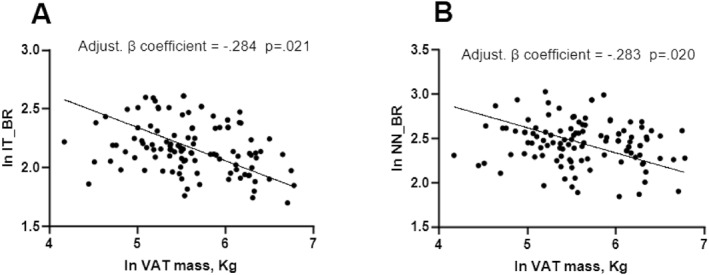
(A, B). Multivariate regressions between VAT Mass and HSA‐derived parameters. Dependent variables: IT_BR (A) and NN_BR (B) Variables tested in the model: total lean%, age, gender, BMI, HbA1c%, physical activity. Factors associated with markers of bone health at a conservative *p*‐value < 0.1 were retained in the model. BR, buckling ratio; IT, Intertrochanteric site; NN, narrow neck site; VAT Mass, Visceral adipose tissue Mass.

In Figures 1 and 2, the significant associations between adiposity markers (Total Fat % and VAT mass, respectively) and HSA‐derived bone geometry parameters adjusted for confounders (Total Lean %, age, sex, BMI, HbA1c% and physical activity) are expressed. In particular, at the intertrochanteric site, Total Fat % was negatively associated with CSMI (β coefficient = −0.429, *p* = 0.0020, Figure 1A) and Z (β coefficient = −0.361, *p* = 0.041, Figure 1B), but not with CSA and BR. At the femur shaft site, Total Fat% was negatively associated with CSA (β coefficient = −0.307, *p* = 0.0051. Figure 1C), CSMI (β coefficient = −0.408, *p* = 0.0073, Figure 1D), Z (β coefficient = −0.415, *p* = 0.0082, Figure 1E), but not with BR. At the narrow neck site, Total Fat % was negatively associated with CSMI (β coefficient = −0.360, *p* = 0.042, Figure 1F), BR (β coefficient = −0.351, *p* = 0.010, Figure 1G) and Z (β coefficient = −0.240, *p* = 0.030, Figure 1H).

Similarly, VAT mass showed an inverse association with BR at the intertrochanteric site (β coefficient = −0.284, *p* = 0.021) and at the narrow neck site (β coefficient = −0.283, *p* = 0.020) (Figure 2A–B).

Total lean % was positively associated with CSA, CSMI and Z at the intertrochanteric and femur shaft sites in the multivariate regression including VAT mass (Table S4) but not in those including Total Fat % (Table S2). BMI was directly associated with CSA and CSMI but inversely with BR at different sites (Tables S2 and S4). Male sex was a positive predictor of several HSA‐derived bone geometry parameters (Tables S2 and S4). Age was inversely associated with CSA and the intertrochanteric and narrow neck sites (Table S4). Finally, HbA1c% and physical activity were not significantly associated with HSA‐derived parameters.

## 4 Discussion

In this study, we show that differences in BMD and hip strength exist among people with AD with and without OW. We further show that fat mass excess, investigated by DXA, is associated with lower bone strength at the hip sites, but not with BMD, independently from confounders such as lean mass, age, sex, HbA1c%, BMI, and physical activity habit. Specifically, we reported that OW is associated with increased BMD at the total hip and left femur neck in both women and men. This finding is consistent with the so‐called ‘obesity paradox’ [44], also suggested by previous studies conducted in people without diabetes, showing that higher BMI is associated with higher BMD [45, 46, 47]. Consistently, also in our population of people with AD, BMI was a significant and constant positive predictor for BMD, independently from confounders, as observed in the general population [45, 46, 47]. One mechanism to explain the higher BMD found in patients with obesity is the higher mechanical loading due to higher body weight [44, 48, 49]. Moreover, we found that vitamin D serum levels were significantly lower in men with obesity compared with those with normal weight. This is consistent with what is well known in the literature, namely that vitamin D levels are lower in patients with obesity due to the larger volume of distribution, the tight binding of vitamin D within fatty tissues, reduced absorption, and diets low in vitamin D [32].

However, it is now evident that obesity is also characterised by abnormalities in bone strength and geometry, which might instead increase the risk of bone fractures [47]. Therefore, BMD alone might not be a reliable index of bone health in the context of obesity. In this regard, HSA parameters at femur sites were proved to be able to predict the occurrence of hip fractures [50, 51, 52], even independently from BMD and other confounders [50].

Accordingly, in this study, we also evaluated HSA‐derived parameters of bone geometry and strength, including CSA, CSMI, BR and Z, in different femur sites (IT, NN, FS). Similar to BMD, CSA and CSMI were found to be higher in different femur sites among people with OW compared with NW. Nonetheless, BMI was positively associated with CSA and CSMI at different femur sites independent of confounders. This trend was previously described in non‐diabetic populations [53, 54], and it might be mediated as well by the increased mechanical loading [44, 48, 49].

However, when we evaluated the impact of adiposity markers on bone health, we found that, differently from BMI, Total Fat% was negatively associated with several parameters of bone geometry and strength (CSMI and Z at intertrochanteric site, CSA, CSMI and Z at femur shaft site and CSMI, BR and Z at narrow neck site), independent of confounders, such as age, lean mass, HbA1c, sex and physical activity habit. This suggests that in people with AD, a worse body composition, with a relative increase in fat mass, is associated with poorer bone quality and strength independently from BMI and other confounders. In addition to fat mass alone, we reported that VAT mass alone could also represent a negative predictor for BR at intertrochanteric and narrow neck sites independent of confounders. Different mechanisms might explain the association between excess fat mass and bone health. First, although it is known that higher BMI is associated with higher BMD, it has also been reported that lean mass is more strongly associated with BMD than fat mass [55] and increased intramuscular fat might blunt the positive association between BMI and BMD [56]. Second, pro‐inflammatory adipokines [32], as well as excessive FFAs [33], which are mostly released by visceral adipose tissue, could impair bone remodelling. Finally, increased marrow adipose tissue has also been proposed as an emerging factor with detrimental effects on bone health and strength [34].

In our study, physical activity was not associated with bone markers, whereas lean mass was positively associated with CSA, CSMI and Z at IT and FS sites, after adjustment by confounders. The literature consistently shows a strong relationship between muscle mass and bone, and several myokines such as IL‐6, irisin, and IGF‐1 exert anabolic and catabolic effects on bone, while the osteokines osteocalcin and sclerostin have been shown to induce muscle anabolism and catabolism, respectively [57]. Although in our study the physical activity data are limited by being self‐reported, adiposity markers nevertheless appear to play a predominant role in our population with AD.

Similarly, poor glucose control, insulin deficiency and increased glucotoxicity and AGEs represent known factors for bone frailty in patients with diabetes [58]. However, in our study, glucose control evaluated with the measurement of the last HbA1c% did not represent a significant predictor of bone health. To note, it is possible that the cross‐sectional evaluation of Hba1C% does not fully grasp the impact of long‐term glucose control on bone health in the study population. Despite this limitation, body composition seems to play a much more significant role in determining bone health in people with AD.

Our study must be read in the light of some limitations, such as: (1) the cross‐sectional design, which does not allow to infer causal‐effect conclusions; (2) the lack of control groups without AD; (3) the limited sample size; (4) Finally, HSA analysis represents an indirect measurement of bone strength, relying on assumptions about the symmetry and shape of bone cross‐sections [34]. Nonetheless, to the best of our knowledge, this is the first study assessing the impact of obesity and fat mass on DXA‐derived parameters of bone health in people with AD. Moreover, we did not limit our evaluations to BMI and BMD, but we extended our analysis to parameters of bone geometry and strength as well as body composition.

In conclusion, this study shows that, although OW is associated with higher BMD, an increase in fat mass is associated with abnormal HSA‐derived parameters of bone geometry and strength in people with AD. Considering the increasing prevalence of OW among people with AD, this study highlights relevant issues about the detrimental effects of fat mass excess on bone health in people with AD.

## Author Contributions

R.R.: data analysis and interpretation, manuscript draft. R.A., F.D.V., F.B., L.D., M.W., D.L., A.L.P., L.D.O.: investigation, data acquisition and curation. R.B.: final revision of the manuscript. E.M.: study design and conceptualization, final revision of the manuscript.

## Funding

Funded by European Union‐Next Generation EU, Mission 4, Component 1, CUP B53D23021820001, Italian Ministry of University and Research, PRIN project #2022NS7PRM.

## Conflicts of Interest

The authors declare no conflicts of interest.

## Supporting information


**Table S1:** Linear regressions testing the associations between Total Fat %, Total lean %, BMI, HbA1c, sex, age, physical activity (independent variables) and total hip, femur neck, and lumbar spine BMD, TBS (dependent variables). Data have been appropriately transformed into natural logarithms. The weight of independent variables on dependent variables is expressed as adjusted *β* coefficient. Abbreviations: BMI, body mass index; BMD, bone mineral density; TBS, trabecular bone score; HbA1c, Haemoglobin A1C. ***p value < 0.001; **p value < 0.01; *p value < 0.05.

**Table S2:** Linear regressions testing the associations between Total Fat %, Total lean %, BMI, HbA1c, sex, age, physical activity (independent variables) and CSA, CSMI, BR and Z at IT, FS, and NN sites (dependant variables). Data have been appropriately transformed into natural logarithms. The weight of independent variables on dependent variables is expressed as adjusted *β* coefficient. Abbreviations: BMI, body mass index; HbA1c, Haemoglobin A1C; CSA, cross sectional area; CSMI, cross sectional moment of inertia; BR, buckling ration; Z, section modules; IT, intertrochanteric site; FS, femur shaft site; NN, narrow neck site. ***p value < 0.001; **p value < 0.01; *p value < 0.05.

**Table S3:** Linear regressions testing the associations between VAT Mass, Total lean %, BMI, HbA1c, sex, age, physical activity (independent variables) and total hip, femur neck, and lumbar spine BMD and TBS (dependant variables). Data have been appropriately transformed into natural logarithms. The weight of independent variables on dependent variables is expressed as adjusted *β* coefficient. Abbreviations: VAT Mass, visceral adipose tissue mass; BMI, body mass index; BMD, bone mineral density; TBS, trabecular bone score; HbA1c, Haemoglobin A1C. ***p value < 0.001; **p value < 0.01; *p value < 0.05.

**Table S4:** Linear regressions testing the associations between VAT mass, Total lean %, BMI, HbA1c, sex, age, physical activity (independent variables) and CSA, CSMI, BR and Z at IT, FS, and NN sites (dependant variables). Data have been appropriately transformed into natural logarithms. The weight of independent variables on dependent variables is expressed as adjusted *β* coefficient. Abbreviations: VAT Mass, visceral adipose tissue mass; BMI, body mass index; HbA1c, Haemoglobin A1C; CSA, cross sectional area; CSMI, cross sectional moment of inertia; BR, buckling ration; Z, section modules; IT, intertrochanteric site; FS, femur shaft site; NN, narrow neck site. ***p value < 0.001; **p value < 0.01; *p value < 0.05.

## Data Availability

The data that support the findings of this study are available from the corresponding author upon reasonable request.
