# Supplementary material for: Fat Mass Influences Femur Bone Strength and Geometry Parameters, but Not Bone Mineral Density, in Autoimmune Diabetes: A Pilot Study
Source: Diabetes Metab Res Rev. 2026 Mar 19;42(3):e70149. doi: 10.1002/dmrr.70149 (PMC13000683; doi:10.1002/dmrr.70149)
Supplement: Supplementary file 1 — Table S1: Linear regressions testing the associations between Total Fat %, Total lean %, BMI, HbA1c, sex, age, physical activity (independent variables) and total hip, femur neck, and lumbar spine BMD, TBS (dependent variables). Data have been appropriately transformed into natural logarithms. The weight of independent variables on dependent variables is expressed as adjusted β coefficient. Abbreviations: BMI, body mass index; BMD, bone mineral density; TBS, trabecular bone score; HbA1c, Haemoglobin A1C. ***p value < 0.001; **p value < 0.01; *p value < 0.05. [file DMRR-42-e70149-s003.docx]

|  | **ln Total Hip BMD** | **ln Femur Neck BMD** | **ln Lumbar Spine BMD** | **TBS** |
| --- | --- | --- | --- | --- |
| **ln Total**  **Fat%** | 0.029 | 0.119 | -0.388 | -0.366 |
| **ln Total Lean%** | 0.113 | 0.135 | -0.068 | -0.076 |
| **ln BMI, kg/m2** | **0.605***** | **0.456***** | **0.652***** | 0.193 |
| **ln HbA1c %** | -0.096 | -0.041 | -0.015 | -0.143 |
| **Sex,**  **F=1 M=2** | **0.225*** | 0.218 | -0.051 | **-0.359*** |
| **ln age, years** | -0.146 | **-0.202*** | -0.107 | **-0.456*** |
| **physically active, yes=1 no=0** | -0.049 | 0.009 | -0.090 | -0.028 |

**Table S1. Linear regressions testing the associations between Total Fat %, Total lean %, BMI, HbA1c, sex, age, physical activity (independent variables) and Total Hip, Femur Neck and Lumbar Spine BMD, TBS (dependant variables).** Data have been appropriately transformed in natural logarithms. The weight of independent variables on dependant variables is expressed as adjusted β coefficient.
Abbreviations: BMI, body mass index; BMD, bone mineral density; TBS, trabecular bone score; HbA1c, Haemoglobin A1C

*** p value <0.001; ** p value <0.01; * p value <0.05
