# Supplementary material for: Fat Mass Influences Femur Bone Strength and Geometry Parameters, but Not Bone Mineral Density, in Autoimmune Diabetes: A Pilot Study
Source: Diabetes Metab Res Rev. 2026 Mar 19;42(3):e70149. doi: 10.1002/dmrr.70149 (PMC13000683; doi:10.1002/dmrr.70149)
Supplement: Supplementary file 2 — Table S2: Linear regressions testing the associations between Total Fat %, Total lean %, BMI, HbA1c, sex, age, physical activity (independent variables) and CSA, CSMI, BR and Z at IT, FS, and NN sites (dependant variables). Data have been appropriately transformed into natural logarithms. The weight of independent variables on dependent variables is expressed as adjusted β coefficient. Abbreviations: BMI, body mass index; HbA1c, Haemoglobin A1C; CSA, cross sectional area; CSMI, cross sectional moment of inertia; BR, buckling ration; Z, section modules; IT, intertrochanteric site; FS, femur shaft site; NN, narrow neck site. ***p value < 0.001; **p value < 0.01; *p value < 0.05. [file DMRR-42-e70149-s001.docx]

|  | **ln IT_CSA** | **ln IT_CSMI** | **ln IT_BR** | **ln IT_Z** | **ln FS_CSA** | **ln FS_CSMI** | | **ln FS_BR** | **ln FS_Z** | **ln NN_CSA** | **ln NN_CSMI** | **ln NN_BR** | **ln NN_Z** |
| --- | --- | --- | --- | --- | --- | --- | --- | --- | --- | --- | --- | --- | --- |
| **ln Total Fat%** | -0.217 | **-0.429**** | -0.199 | **-0.361*** | **-0.307**** | **-0.408**** | -0.049 | | **-0.414**** | -0.129 | **-0.360*** | **-0.351*** | **-0.240*** |
| **ln Total**  **Lean%** | 0.174 | 0.121 | -0.176 | 0.124 | 0.118 | 0.100 | -0.075 | | 0.118 | 0.094 | -0.030 | -0.218 | 0.026 |
| **ln BMI, kg/m2** | **0.142***** | **0.555***** | **-0.369**** | **0.582***** | **0.645***** | **0.524***** | **-0.381**** | | **0.595***** | **0.502***** | **0.394***** | **-0.322*** | **0.440***** |
| **ln HbA1c,%** | -0.043 | 0.295 | 0.127 | 0.245 | -0.014 | 0.059 | 0.244 | | 0.074 | -0.002 | 0.023 | 0.126 | -0.029 |
| **Sex, F=1 M=2** | 0.106 | 0.145 | -0.073 | 0.086 | **0.346***** | **0.311*** | -0.086 | | **0.322**** | **0.349**** | **0.460**** | 0.007 | **0.447***** |
| **ln age, years** | 0.091 | 0.134 | 0.087 | -0.022 | -0.351 | 0.116 | 0.207 | | 0.132 | -0.109 | 0.077 | 0.341 | -0.029 |
| **Physically active, yes=1 no=0** | -0.062 | -0.051 | 0.129 | 0.065 | -0.044 | -0.033 | -0.001 | | -0.033 | 0.028 | 0.067 | 0.064 | 0.039 |

**Table S2. Linear regressions testing the associations between Total Fat %, Total lean %, BMI, HbA1c, sex, age, physical activity (independent variables) and CSA, CSMI, BR and Z at IT , FS, NN sites (dependant variables).** Data have been appropriately transformed in natural logarithms. The weight of independent variables on dependant variables is expressed as adjusted β coefficient.
Abbreviations: BMI, body mass index; HbA1c, Haemoglobin A1C; CSA, cross sectional area; CSMI, cross sectional moment of inertia; BR, buckling ration; Z, section modules; IT, intertrochanteric site; FS, femur shaft site; NN, narrow neck site.
*** p value <0.001; ** p value <0.01; * p value <0.05.
