# Supplementary material for: Fat Mass Influences Femur Bone Strength and Geometry Parameters, but Not Bone Mineral Density, in Autoimmune Diabetes: A Pilot Study
Source: Diabetes Metab Res Rev. 2026 Mar 19;42(3):e70149. doi: 10.1002/dmrr.70149 (PMC13000683; doi:10.1002/dmrr.70149)
Supplement: Supplementary file 4 — Table S4: Linear regressions testing the associations between VAT mass, Total lean %, BMI, HbA1c, sex, age, physical activity (independent variables) and CSA, CSMI, BR and Z at IT, FS, and NN sites (dependant variables). Data have been appropriately transformed into natural logarithms. The weight of independent variables on dependent variables is expressed as adjusted β coefficient. Abbreviations: VAT Mass, visceral adipose tissue mass; BMI, body mass index; HbA1c, Haemoglobin A1C; CSA, cross sectional area; CSMI, cross sectional moment of inertia; BR, buckling ration; Z, section modules; IT, intertrochanteric site; FS, femur shaft site; NN, narrow neck site. ***p value < 0.001; **p value < 0.01; *p value < 0.05. [file DMRR-42-e70149-s002.docx]

|  | **ln IT_CSA** | **ln IT_CSMI** | **ln IT_BR** | **ln IT_Z** | **ln FS_CSA** | **ln FS_CSMI** | | **ln FS_BR** | **ln FS_Z** | **ln NN_CSA** | **ln NN_CSMI** | **ln NN_BR** | **ln NN_Z** |
| --- | --- | --- | --- | --- | --- | --- | --- | --- | --- | --- | --- | --- | --- |
| **ln VAT Mass, kg** | 0.206 | 0**.**072 | **-0.284*** | 0.137 | 0.082 | 0.167 | 0.053 | | 0.128 | 0.255 | 0.168 | **-0.283*** | 0.236 |
| **ln Total**  **Lean%** | **0.279*** | **0.295*** | -0.127 | **0.279*** | **0.249**** | **0.257*** | -0.120 | | **0.292**** | 0.170 | 0.127 | -0.110 | 0.143 |
| **ln BMI, kg/m2** | **0.349**** | **0.371**** | **-0.287*** | **0.387**** | **0.505***** | **0.295*** | **-0.460**** | | **0.388***** | **0.324**** | 0.183 | **-0.293*** | **0.235*** |
| **ln HbA1c,%** | -0.054 | -0.024 | 0.124 | -0.046 | -0.009 | 0.004 | 0.015 | | 0.057 | -0.010 | 0.008 | 0.117 | -0.041 |
| **Sex, F=1 M=2** | 0.173 | **0.321*** | 0.180 | **0.252*** | **0.411***** | **0.360*** | **-0.136** | | **0.427**** | **0.256*** | **0.517***** | **0.324*** | **0.413**** |
| **ln age, years** | **-0.260*** | -0.101 | 0.399 | -0.119 | -0.063 | 0.040 | 0.218 | | 0.034 | **-0.239*** | -0.035 | 0.438 | -0.160 |
| **Physically active, yes=1 no=0** | -0.079 | -0.028 | 0.147 | -0.071 | -0.065 | -0.068 | 0.034 | | -0.046 | 0.009 | 0.052 | 0.080 | 0.020 |

**Table S4. Linear regressions testing the associations between VAT mass, Total lean %, BMI, HbA1c, sex, age, physical activity (independent variables) and CSA, CSMI, BR and Z at IT, FS, NN sites (dependant variables).** Data have been appropriately transformed in natural logarithms. The weight of independent variables on dependant variables is expressed as adjusted β coefficient.
Abbreviations: VAT Mass, visceral adipose tissue mass; BMI, body mass index; HbA1c, Haemoglobin A1C; CSA, cross sectional area; CSMI, cross sectional moment of inertia; BR, buckling ration; Z, section modules; IT, intertrochanteric site; FS, femur shaft site; NN, narrow neck site.
*** p value <0.001; ** p value <0.01; * p value <0.05.
